# Chlamydia prevention and management in Australia: reducing the burden of disease

**DOI:** 10.5694/mja2.51749

**Published:** 2022-11-06

**Authors:** Stephanie C Munari, Jane L Goller, Margaret E Hellard, Jane S Hocking

**Affiliations:** ^1^ Burnet Institute Melbourne VIC; ^2^ University of Melbourne Melbourne VIC; ^3^ Alfred Hospital Melbourne VIC

**Keywords:** Chlamydia infections, Communicable diseases, Sexually transmitted diseases, Disease transmission infectious, Public health, Prevention and control

To reduce the burden of chlamydia in Australia, comprehensive patient and partner management is required

Fourteen years on from Hocking and colleagues’^1^ call for innovative *Chlamydia trachomatis* (hereafter referred to as chlamydia) screening programs to reduce the high rates of notifications in Australia at the time, chlamydia remains as the country's most notified bacterial sexually transmissible infection (STI). From 2014 to 2018, the overall number and rate of chlamydia notifications rose by 21% and 15% respectively.^2^ Most new chlamydia infections are occurring among young people aged 15–29 years, and in 2019, women aged 15–24 years old recorded higher notification rates per 100 000 population compared with similarly aged men. An important exception is that notification rates appear to be falling in women under 25 years old,^3^ for whom chlamydia testing rates have plateaued and positivity among those tested is declining.^4^ In addition to people with female reproductive organs and young people aged 15–29 years, chlamydia is also disproportionately high among Aboriginal and Torres Strait Islander people, people living in remote and very remote areas, those with greater socio‐economic disadvantage,^5^ and among gay and bisexual men.^6^ People who are pregnant are also a priority population, where chlamydia infection is associated with miscarriage, stillbirth, preterm birth, low birth weight, and postpartum infections in the mother and/or newborn.^7^, ^8^ Once treated, an individual may become reinfected, contributing to further potential transmission and increasing the risk of morbidity in the form of pelvic inflammatory disease (PID), ectopic pregnancies, infertility, and chronic pelvic pain.^9^ Chlamydia remains a significant public health issue in Australia, with the search for novel prevention and management strategies ongoing.

Current Australian guidelines recommend testing for chlamydia in people aged under 30 years who are sexually active, pregnant, at increased risk of complications, presenting with a clinical picture suggestive of chlamydia infection, requesting an STI check, and those who have had a partner change or STI in the past 12 months or a sexual partner with an STI.^10^ For those who test positive, retesting is recommended at 3 months after treatment to identify possible reinfection.^10^ Tests can be self‐collected by the patient, and the treatment of a positive case consists of a 7‐day course of doxycycline as first line therapy or a single dose of azithromycin.^10^ So with straightforward testing and treatment, what are some of the gaps when it comes to chlamydia prevention and management in Australia, and what needs to be done to reduce the associated morbidity?

## Improve chlamydia retesting

One gap is the low rates of chlamydia retesting after treatment for a prior infection, along with high reinfection rates, increasing the potential for onward transmission and reproductive complications. Chlamydia reinfection rates among young women have been found to be high, with a calculated cumulative risk of reinfection of 20.3% (95% CI, 11.6–31.7%) among women aged 16–25 years in Australia.^11^ In a study among 16–29‐year‐old women attending 25 general practice clinics in Australia between 2008 and 2009, only 70 out of 175 individuals (40%) were retested within 1.5–12 months of a positive test.^12^ Further research among 2357 individuals of the same age group attending 128 regional general practices between 2010 and 2015 found that only 26.5% of individuals were retested within 6 weeks to 6 months following a previously positive chlamydia test, with 11.9% having a positive retest result.^13^

Despite insufficient evidence to demonstrate the effect of repeat chlamydia testing after treatment on chlamydia transmission rates, mathematical modelling suggests that earlier testing and retesting at the recommended 3 months after diagnosis may increase the window of opportunity for treatment and thus reduce the likelihood of reproductive complications.^14^

## Improve diagnosis of pelvic inflammatory disease

Another gap in chlamydia management is the underdiagnosis of PID in primary care settings. The population‐attributable fraction of chlamydia infection in PID is estimated to be around 20–30%.^15^ If untreated, around 17% of chlamydia infections in women will progress to PID,^16^ with the risk of PID increasing by 20% with each repeat chlamydia infection.^9^ PID is predominantly diagnosed according to clinical signs and symptoms; however, clinical diagnosis can be challenging due to the diverse range of clinical presentations. A more definitive investigation such as laparoscopy is considered a more invasive and resource‐intensive test, and usually not requested in general practice unless for more complicated infections with poor treatment response.^17^ Further, many general practitioners cite that the need for a pelvic examination can be a barrier to diagnosis.^17^

## Move away from asymptomatic screening

Another reason for Australia's high chlamydia rates may lie in the approach to detection. Globally, among high income countries, the focus of chlamydia control measures is shifting away from the promotion of asymptomatic screening towards improved case management to reduce the complications of infections.^18^ Currently, there is insufficient evidence to show that asymptomatic chlamydia screening in women reduces chlamydia‐associated infertility, due to challenges in identifying tubal infertility and tying this to a previous chlamydia infection.^19^ Findings from the ACCEPt trial — a large cluster randomised controlled trial published in 2018 — also demonstrated that targeted and opportunistic chlamydia testing in primary care may not contribute to significant reductions in prevalence.^20^ Critically, the trial demonstrated that despite significant efforts, it is difficult to achieve testing levels high enough to have an impact on prevalence.^20^

Consideration must also be given to the possible harms from asymptomatic screening, including the increasing antimicrobial resistance due to the inappropriate use, and overuse, of antibiotics, psychological distress associated with false positive diagnoses, and adverse impacts on microbiota.^19^ The United Kingdom, the Netherlands and Sweden are all re‐evaluating their chlamydia control strategies, with a move away from recommending widespread opportunistic chlamydia screening towards a focus on the management of diagnosed cases to prevent ongoing transmission and complications.^19^ If Australia is to reduce its burden of disease from chlamydia, an increased focus on case and partner management will be required.

## Enhance partner notification and management

Integrating timely testing, retesting and partner management into routine care has been identified as a national priority to enhance chlamydia case and contact management in Australia.^21^

In addition to primary prevention strategies such as sexual health education, notifying, testing and treating sexual partners from the previous 6 months can also help to interrupt ongoing transmission and reduce the risk of reinfection and complications.^18^ GPs are well placed to provide guidance for patients when engaging in partner notification, including through the use of anonymous online notification tools. Moreover, patient‐delivered partner therapy is an effective way to both treat the partners and reduce reinfection in the index case^22^, ^23^ — the Australasian Contact Tracing Guidelines detail the situations where patient‐delivered partner therapy is appropriate.^10^

## Embrace new testing approaches

Innovative testing strategies might also improve the detection and management of chlamydia, particularly among young people in Australia. Augmented by the coronavirus disease 2019 (COVID‐19) pandemic, the increasing use of digital health technology offers an enticing opportunity for reaching a population with high technical literacy skills. Online primary care consultations that use telehealth, the increasing use of home sampling kits for posting back to a laboratory, electronic prescriptions, and trials of online sexual health hubs might help overcome identified barriers to accessing traditional sexual health service delivery, including concerns about privacy, confidentiality, and perceived stigma.^24^ Online services can also offer increased accessibility and convenience for young people who often have many competing life priorities.

Improving the delivery of sexual health services through quality improvement programs, including better access to testing and case management, has also been shown to be effective in increasing STI test uptake in primary health care settings in remote Australian Aboriginal communities.^25^

## Next steps

To reduce the burden of disease from chlamydia in Australia, comprehensive follow‐up of cases and contacts to reduce the risk of complications is required. When chlamydia is detected, retesting at 3 months for reinfection and performing thorough partner tracing and management can help interrupt transmission and reduce the risk of reinfection and reproductive complications. Further studies investigating the timing of testing and treatment of chlamydia infections on the progression to reproductive complications will help guide public health strategies to further reduce the burden of chlamydia in Australia.

## Open access

Open access publishing facilitated by The University of Melbourne, as part of the Wiley ‐ The University of Melbourne agreement via the Council of Australian University Librarians.

## Competing interests

Margaret Hellard receives funding from Gilead Science and Abbvie for investigator‐initiated research unrelated to this area of work.

## Provenance

Not commissioned; externally peer reviewed.

## References

[1] Hocking JS , Walker J , Regan D , et al. Chlamydia screening ‐ Australia should strive to achieve what others have not. Med J Aust 2008; 188: 106‐168. https://www.mja.com.au/journal/2008/188/2/chlamydia‐screening‐australia‐should‐strive‐achieve‐what‐others‐have‐not 18205585 10.5694/j.1326-5377.2008.tb01533.x

[2] Kirby Institute . National update on HIV, viral hepatitis and sexually transmissible infections in Australia: 2009–2018. https://kirby.unsw.edu.au/report/national‐update‐hiv‐viral‐hepatitis‐and‐sexually‐transmissible‐infections‐australia‐2009‐2018 (viewed Apr 2022).

[3] Kirby Institute . Sexually transmissible infections [website]. https://data.kirby.unsw.edu.au/STIs (viewed Apr 2022).

[4] Bourchier L , Malta S , Temple‐Smith M , Hocking J . Do we need to worry about sexually transmissible infections (STIs) in older women in Australia? An investigation of STI trends between 2000 and 2018. Sex Health 2020; 17: 517‐524. 33334416 10.1071/SH20130

[5] Gibney KB , Cheng AC , Hall R , Leder K . Sociodemographic and geographical inequalities in notifiable infectious diseases in Australia: a retrospective analysis of 21 years of national disease surveillance data. Lancet Infect Dis 2017; 17: 86‐97. 27789179 10.1016/S1473-3099(16)30309-7

[6] Kirby Institute . HIV, viral hepatitis and sexually transmissible infections in Australia: annual surveillance report 2018. https://kirby.unsw.edu.au/report/asr2018 (viewed Sept 2022).

[7] Olson‐Chen C , Balaram K , Hackney DN . Chlamydia trachomatis and adverse pregnancy outcomes: meta‐analysis of patients with and without infection. Matern Child Health J 2018; 22: 812‐821. 29417367 10.1007/s10995-018-2451-z

[8] Tang W , Mao J , Li KT , et al. Pregnancy and fertility‐related adverse outcomes associated with Chlamydia trachomatis infection: a global systematic review and meta‐analysis. Sex Transm Infect 2020; 96: 322‐329. 31836678 10.1136/sextrans-2019-053999 PMC7292777

[9] Davies B , Ward H , Leung S , et al. Heterogeneity in risk of pelvic inflammatory diseases after chlamydia infection: a population‐based study in Manitoba, Canada. J Infect Dis 2014; 210 (Suppl): S549‐S555. 25381374 10.1093/infdis/jiu483 PMC4231643

[10] Australasian Society for HIV, Viral Hepatitis and Sexual Health Medicine. Australian STI Management Guidelines for Use in Primary Care: chlamydia. ASHM, 2021. https://sti.guidelines.org.au/sexually‐transmissible‐infections/chlamydia/ (viewed Mar 2022).

[11] Walker J , Tabrizi SN , Fairley CK , et al. Chlamydia trachomatis incidence and re‐infection among young women ‐ behavioural and microbiological characteristics. PloS One 2012; 7: e37778. 22662220 10.1371/journal.pone.0037778 PMC3360595

[12] Bowring AL , Gouillou M , Guy R , et al. Missed opportunities ‐ low levels of chlamydia retesting at Australian general practices, 2008–2009. Sex Transm Infect 2012; 88: 330‐334. 22406524 10.1136/sextrans-2011-050422

[13] Huang Y , Hocking J , Braat S , Goller J . P142 Low retesting and high reinfection rates among young people treated for chlamydia in Australian general practices. Sex Transm Infect 2021; 97: A96.

[14] Herzog SA , Althaus CL , Heijne JC , et al. Timing of progression from Chlamydia trachomatis infection to pelvic inflammatory disease: a mathematical modelling study. BMC Infect Dis 2012; 12: 187. 22883325 10.1186/1471-2334-12-187 PMC3505463

[15] van Bergen JEAM , Hoenderboom BM , David S , et al. Where to go to in chlamydia control? From infection control towards infectious disease control. Sex Transm Infect 2021; 97: 501‐506. 34045364 10.1136/sextrans-2021-054992 PMC8543211

[16] Price MJ , Ades AE , Soldan K , et al. The natural history of *Chlamydia trachomatis* infection in women: a multi‐parameter evidence synthesis. Health Technol Assess 2016; 20: 1‐250. 10.3310/hta20220 PMC4819202 27007215

[17] Bittleston H , Coombe J , Temple‐Smith M , et al. Diagnosis of pelvic inflammatory disease and barriers to conducting pelvic examinations in Australian general practice: findings from an online survey. Sex Health 2021; 18: 180‐186. 33832551 10.1071/SH20176

[18] Unemo M , Bradshaw CS , Hocking JS , et al. Sexually transmitted infections: challenges ahead. Lancet Infect Dis 2017; 17: e235‐e279. 28701272 10.1016/S1473-3099(17)30310-9

[19] Low N , Hocking JS , van Bergen J . The changing landscape of chlamydia control strategies. Lancet 2021; 398: 1386‐1388. 34562393 10.1016/S0140-6736(21)02002-X

[20] Hocking JS , Temple‐Smith M , Guy R , et al. Population effectiveness of opportunistic chlamydia testing in primary care in Australia: a cluster‐randomised controlled trial. Lancet 2018; 392: 1413‐1422. 30343857 10.1016/S0140-6736(18)31816-6

[21] Australian Government Department of Health . Fourth National Sexually Transmissible Infections Strategy 2018–2022. https://www.health.gov.au/resources/publications/fourth‐national‐sexually‐transmissible‐infections‐strategy‐2018‐2022 (viewed Sept 2022).

[22] Althaus CL , Turner KM , Mercer CH , et al. Effectiveness and cost‐effectiveness of traditional and new partner notification technologies for curable sexually transmitted infections: observational study, systematic reviews and mathematical modelling. Health Technol Assess 2014; 18: 1‐100. 10.3310/hta18020 PMC4780998 24411488

[23] Coombe J , Goller J , Bittleston H , et al. Patient‐delivered partner therapy: one option for management of sexual partner(s) of a patient diagnosed with a chlamydia infection. Aust J Gen Pract 2022; 51: 425‐429. 35637588 10.31128/AJGP-07-21-6066

[24] Baxter S , Blank L , Guillaume L , et al. Views of contraceptive service delivery to young people in the UK: a systematic review and thematic synthesis. J Fam Plann Reprod Health Care 2011; 37: 71‐84. 21454259 10.1136/jfprhc.2010.0014

[25] Ward J , Guy RJ , Rumbold AR , et al. Strategies to improve control of sexually transmissible infections in remote Australian Aboriginal communities: a stepped‐wedge, cluster‐randomised trial. Lancet Glob Health 2019; 7: e1553‐e1563. 31607467 10.1016/S2214-109X(19)30411-5

